# A Reproducible Protocol for the Isolation of Malaria-Derived Extracellular Vesicles by Differential Centrifugation

**DOI:** 10.3390/mps7060092

**Published:** 2024-11-09

**Authors:** Tosin Opadokun, Petra Rohrbach

**Affiliations:** Institute of Parasitology, McGill University, Sainte-Anne-de-Bellevue, QC H9X 3V9, Canada; tosin.opadokun@mcgill.ca

**Keywords:** *Plasmodium falciparum*, malaria, culture, extracellular vesicles, isolation, differential centrifugation, Western blot analysis, markers

## Abstract

Over the last few decades, malaria-derived extracellular vesicles (EVs) have gained increasing interest due to their role in disease pathophysiology and parasite biology. Unlike other EV research fields, the isolation of malaria EVs is not standardized, hampering inter-study comparisons. Most malaria EV studies isolate vesicles by the “gold-standard” technique of differential (ultra)centrifugation (DC). Here, we describe in detail an optimized and reproducible protocol for the isolation of malaria-derived EVs by DC. The protocol begins with a description of cultivating high-parasitemia, synchronous *P. falciparum* cultures that are the source of EV-containing conditioned culture media. The isolation protocol generates two EV subtypes, and we provide details of characterizing these distinct subtypes by analyzing human and parasite proteins by Western blot analysis. We identify some of these proteins as suitable markers for malaria EV subpopulations and subtypes.

## 1. Introduction

**Figure 1:**
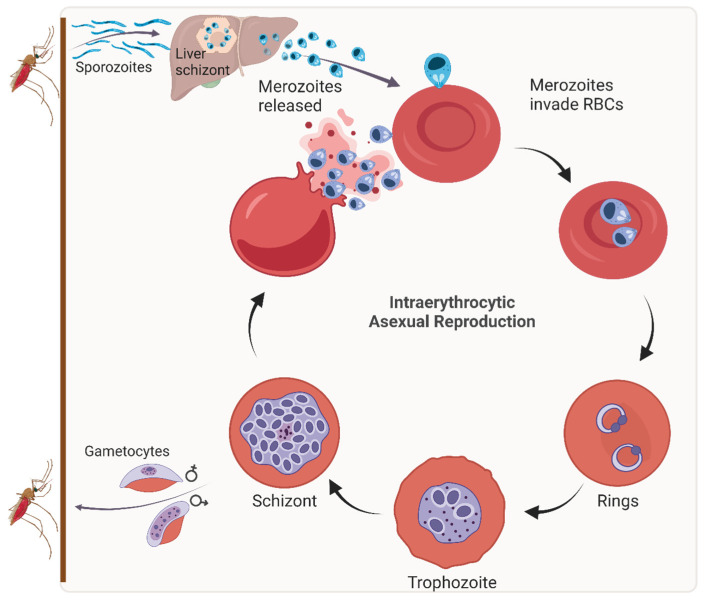
Life cycle of *P. falciparum*. Sporozoites injected into the host during the blood meal of an infected female *Anopheles* mosquito develop into liver schizonts that rupture to release merozoites. Merozoites invade RBCs, where they develop cyclically over the course of 48 h through 3 asexual life stages, namely rings (~24 h), trophozoites (~14 h), and schizonts (~10 h). Repeated rupture of infected RBCs releases toxins and parasite waste products into the bloodstream that induce immune responses, resulting in the clinical manifestation of the disease in infected individuals.

Malaria is a vector-borne protozoan disease, the causative agents of which belong to the parasitic genus *Plasmodium*. *Plasmodium* species possess a complex life cycle involving a mosquito and a vertebrate host; in humans, five species cause malaria, namely *P. falciparum*, *P. vivax*, *P. ovale*, *P. malariae*, and *P. knowlesi* [1,2]. Sexual development occurs in female *Anopheles* mosquitoes that subsequently inoculate malaria parasites into a human during their blood meal (Figure 1). These cparasites (termed sporozoites) migrate to the liver, where they undergo replication to form schizonts. Merozoites released from the liver schizonts infect red blood cells (RBCs), where they undergo cycles of asexual development from young ring stages, to trophozoites and, finally, mature schizonts. For perpetuation of the life cycle, some blood-stage parasites differentiate into sexual blood forms that are taken up by female *Anopheles* mosquitoes during a blood meal. The asexual blood stages of *Plasmodium* spp. are exclusively responsible for the clinical manifestations of malaria, which may be absent, mild, severe, or life-threatening, depending on the age and immune status of the infected individual.

In 2022, there were 249 million malaria cases and 608,000 malaria deaths reported globally, with *P. falciparum* accounting for up to 94% of these statistics [3]. *P. falciparum* causes severe malaria due to the parasite’s unique pathogenic mechanisms that include cytoadherence and sequestration of mature-stage infected RBCs (iRBCs) in the microvasculature of various organs, which is accompanied by an imbalanced host inflammatory response [4,5,6]. There is an increasing number of creports of severe malaria in infections with *P. vivax* [7] and *P. knowlesi* [8], the mechanisms of which differ from *P. falciparum*. Overall, the pathogenic processes involved in severe malaria are not completely understood. Many aspects of the pathophysiology of severe malaria continue to emerge, one of which is the involvement of extracellular vesicles (EVs) [9,10].

**Figure 2:**
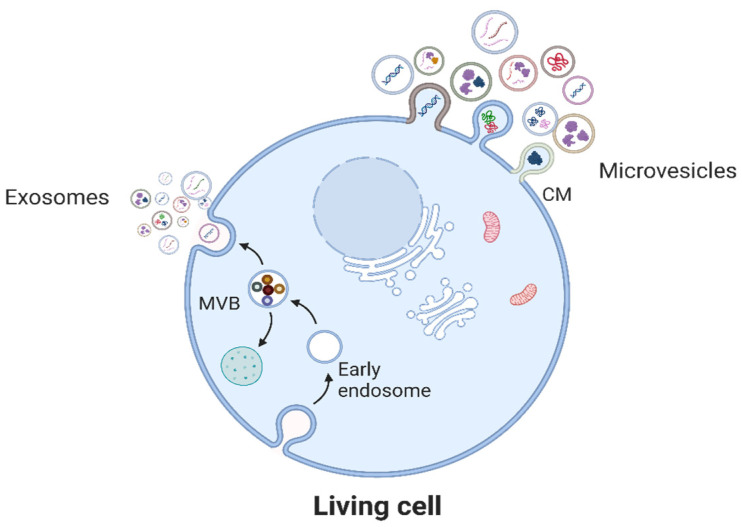
EVs may be generated from viable cells either as microvesicles by directly budding off the cell membrane (CM) or as exosomes by the fusion of multivesicular bodies (MVBs) with the CM. EVs are heterogenous in their size and biomolecular composition.

EVs are small particles (50–1000 nm) that are released by all living cells (Figure 2) and contain active biomolecules (which include nucleic acids, proteins, lipids, and metabolites) bound by a lipid bilayer [11,12]. EVs may be released from cells as exosomes following fusion of multivesicular bodies with the cell membrane, or as microvesicles formed by direct outward budding, also from the cell membrane [11,12]. These vesicles interact with target cells to deliver their biomolecular cargo that is processed by the recipient cells with resultant phenotypic changes [11,12]. In malaria infection, particularly severe malaria, various cells, including the host RBCs of malaria parasites, release elevated levels of EVs with different downstream functions. Studies have shown that *P. falciparum*-infected RBC-derived EVs (*Pf*-iRBC-EVs) are taken up by immune cells [13,14,15,16,17], endothelial cells [18], and other iRBCs [14,19].

The findings of these studies have implicated EVs as central players, not only in the immunopathogenesis of malaria [14,17], but also in the survival and transmission of malaria parasites by inducing drug resistance in otherwise susceptible parasite populations [19] and promoting differentiation of sexual stages of the parasites from asexual stages [14,19]. Furthermore, malaria-derived EVs have been identified as potential drug targets [19], antimalarial drug conveyances [20], and biomarkers for severe disease [10].

The physiological and/or pathological roles of any EV population are defined by their biochemical composition that, alongside their physical properties, are intrinsically and vastly heterogenous across different EV subtypes and subpopulations [21,22,23]. Over the past four decades, there has been a series of functional EV studies in various disciplines that continue to describe diverse functions for EVs [24,25]. Requisites for any functional EV study are EV isolation and characterization [26]. While the International Society for Extracellular Vesicles (ISEV) incessantly advocates for standardized EV research by publishing guidelines for isolation, characterization, functional studies, and data reporting [26,27,28], the standardization, particularly of EV isolation methodologies, remains inadequate [24]. Available EV isolation techniques and methodologies are as heterogenous as EVs themselves, yielding functionally, quantitatively, and qualitatively different EVs from the same starting material (conditioned culture medium, biofluids, or tissues) [29,30,31,32,33]. Consequently, inter-study comparison in the same or similar EV-focused fields is virtually impossible, and consolidating conclusions about EVs drawn from such studies is arduous [24,34].

In the general field of EV research, differential (ultra)centrifugation (DC) and conditioned culture medium (CCM) is the most common EV isolation technique and starting material, respectively [29,35]. This is the case for malaria EV research, particularly for in vitro studies of *Pf*-iRBC-EVs [10,36]. DC is time-consuming and is associated with variable EV yield, EV aggregation, EV disruption, and co-isolation of impurities [37,38,39]. Many studies of malaria EVs include a density gradient in the DC protocol to remove co-isolating contaminants [10]. A DC protocol including density gradient purification is a trade-off of a lower EV yield for higher EV purity that may reduce the amount of measurable EV properties and functions [26]. We have also found this to be true. This supports the argument that, despite the availability of other EV isolation techniques (such as density gradients, immunoaffinity, size exclusion chromatography, precipitation, microfluidics, and commercial kits) that circumvent one or more of the limitations of DC, it remains the preferred technique for EV-containing CCM as it is cost-efficient and suitable for large sample volumes obtained from cell cultures [40]. Moreover, other isolation techniques have their own drawbacks [40] and with an understanding of the basic principles and parameters (such as the nature of the EV-containing starting material, rotor types, rotor k-factors, centrifugation speeds, and centrifugation times), DC protocols can be optimized to improve the yield and purity of EVs [38].

*P. falciparum* is the only human *Plasmodium* specie for which a robust continuous in vitro culture system is available [41,42,43]. A culture of *P. falciparum* comprises a thin layer of stationary or shaking human RBCs infected with the parasite and supported by an overlaying culture medium that is supplemented with nutrients, receives a steady supply of a mixture of gases (oxygen, nitrogen, and carbon dioxide), and is incubated at 37 °C [41,44]. As these cultures contain two eukaryotic cells—host RBCs and the intraerythrocytic parasite, both of which are known to release EVs [45,46,47]—isolated EVs from the CCM plausibly comprise a heterogenous mixture of EVs from uninfected and parasite-infected RBCs that can neither be separated nor distinguished. Awareness of this complex culture matrix necessitates the design of a regimented EV isolation protocol and experimental plan, as well as the consideration of pre-analytical parameters that are general to EV research [26], but more so, unique to malaria EV studies, such as hematocrit, age of RBCs, asexual blood stages, and parasitemia. Here, we describe such a protocol.

Early functional studies of malaria EVs have recently been followed by omics studies to describe their biochemical properties [48,49,50]. Malaria EV research, however, is still in its infancy and, as with other disciplines, will benefit from concurrent advancement in studies of EV functions, biogenesis, biology, and methodology [29]. Considering the popularity that EVs continue to gain for their communicative role within malaria parasite populations, as well as between malaria parasites and host cells, it is imperative that isolation and characterization methodologies and protocols be transparent and standardized to enable reproducibility of significant results, inter-study comparison, and validation of key conclusions.

Using the protocol detailed herein, we isolated EVs from synchronous *P. falciparum* stage-specific iRBC cultures [51]. The protocol proved highly reproducible, allowing us to characterize different EV subpopulations (ring-, trophozoite-, schizont-iRBC EVs) and subtypes (pelleted from different centrifugation speeds, labeled as P1 and P2) with distinct biochemical properties [51]. This protocol provides a transparent description of malaria-derived EVs, their definitions, isolation, characterization, data analysis, and reporting of expected results to promote, improve, and assess inter-study reproducibility and comparability [26].

## 2. Experimental Design

Described here is a DC protocol for the isolation and characterization of EVs derived from high-parasitemia, synchronous *P. falciparum*-infected RBC cultures. This protocol was developed using the 3D7 laboratory strain. As such, we cannot determine the overall outcome and expected results of the protocol using other laboratory strains or laboratory-adapted clinical isolates. The stepwise instructions are easy to follow, although maintaining cultures and harvesting the CCM, which is the starting material for EV isolation, are technically demanding. This is due to the need to maintain high-parasitemia cultures and process large volumes of CCM. We have found that CCM volumes of 100–400 mL from healthy cultures of ≥10% parasitemia yield sufficient EVs for downstream analyses (Appendix A, Table A1). Nevertheless, EV yields may vary quantitatively due to technical variabilities, such as apparatus, (ultra)centrifuges, and operator skills [34].

**Figure 3:**
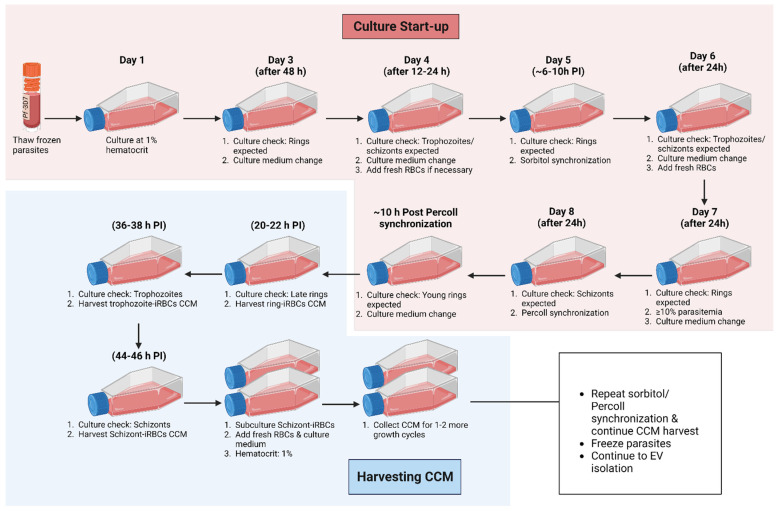
High-parasitemia, synchronous cultures of *P. falciparum* for EV studies can be cultivated within a week of thawing frozen young rings at ≥10% parasitemia, using a combination of sorbitol and Percoll synchronization techniques to enrich ring and mature stages, respectively. To maintain healthy parasites, cultures must be checked, and media must be changed every 24 h. EV-containing CCM from ring-, trophozoite-, and schizont-stage iRBCs can be harvested at 20–22 h, 36–38 h, and 44–46 h post invasion (PI), respectively, for up to 3 invasion cycles, after which synchronization may be repeated to continue CCM harvest and parasites are frozen. CCM = conditioned culture media.

Culture conditions are crucial for the success of this EV isolation protocol. Hence, this protocol begins with a description of starting and maintaining *P. falciparum* cultures for EV studies (Figure 3). Cell cultures of *P. falciparum* iRBCs are started from synchronized young rings previously frozen in glycerin at ≥10% parasitemia. To minimize the confounding effect of non-RBCs, non-RBC EVs, and RBC-derived EVs on the analysis of malaria-derived EVs and interpretation of results, RBCs used in culture are leukoreduced, platelet-reduced, and not older than 14 days of storage. The RBC processing method and storage duration are important and should be reported in any malaria EV study (Appendix A) because (1) residual leukocytes (and platelets) in non-leukoreduced RBC concentrates may release EVs and directly or indirectly induce vesiculation in stored RBCs [52,53], thereby altering the proteome of iRBC EVs. Despite repeated washing of RBCs prior to use in cell cultures to remove contaminating leukocytes and platelets in the buffy coat of non-leukoreduced RBCs, we detected, by immunoblotting [51] and proteomics, non-RBC EV markers in EV isolates from iRBCs and RBCs. This is in line with the findings of a recent EV study in transfusion science [54]. In addition, certain proteins are either markedly elevated or have diminished expression in malaria EVs from cultures of non-leukoreduced blood compared to leukoreduced blood [51]. Leukoreduction of RBCs by centrifugation or filtration almost completely removes white blood cells and platelets, thereby improving RBC quality. Non-leukoreduced RBCs are processed by washing but generally retain white blood cells and platelets [52,53]. (2) RBC vesiculation is a storage lesion that increases significantly with time [55,56], notably from 15 days of storage [57]. (3) From 14 days onward of storage, RBCs become increasingly resistant to merozoite invasion, thereby diminishing parasitemia [58]. As a control, EVs can be isolated from parasite-free RBCs maintained under the same culture conditions as iRBCs and included in downstream analyses to determine baseline vesiculation in uninfected RBCs (uRBCs). This protocol does not use EVs isolated from ionophore-treated RBCs as a control.

Cultures are assessed daily for the parasite life stage and parasitemia by Giemsa staining of a blood smear. The growth medium is changed every 24 h to maintain healthy cultures. The RPMI 1640 growth medium is used, and this is supplemented with AlbuMAX I lipid-rich bovine serum albumin (BSA), which also contributes to attaining high-parasitemia cultures [59]. AlbuMAX I (and AlbuMAX II) is a safe, convenient, and economical replacement for EV-containing human serum. Biological, biochemical, and molecular characteristics of *P. falciparum* may be altered in serum or serum-free cultivation conditions [59,60]. Therefore, in the absence of studies investigating the influence of these conditions on EV release in *P. falciparum* cultures, supplementation with AlbuMAX or serum is an important parameter that should be reported in malaria EV studies (Appendix A, Table A2). If the use of serum is inevitable, a serum-containing non-conditioned culture medium should be included in downstream analyses to control for contaminating serum EVs or non-EV components [26,61]. Alternatively, a serum-containing culture medium may be depleted of EVs prior to use [62].

**Figure 4:**
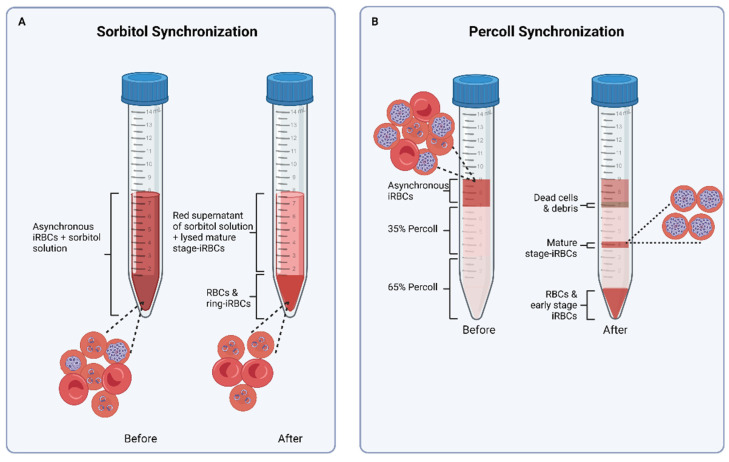
Schematic of synchronization techniques for achieving synchronous high-parasitemia *P. falciparum* cultures for EV isolation. The tubes on the left and right of images A and B represent before and after centrifugation, respectively. (**A**) Asynchronous cultures contain RBCs infected with rings, trophozoites, and schizonts. Sorbitol lyses mature-stage iRBCs, while ring-iRBCs and RBCs are preserved. These cells are separated from the sorbitol after centrifugation (pellet). (**B**) Percoll synchronization recovers mature-stage iRBCs, separating them from uninfected RBCs and early-stage iRBCs after centrifugation.

*P. falciparum* cultures are typically maintained at 4% hematocrit and 5% parasitemia. Hematocrit is the percentage of the volume of RBCs in a culture medium, while parasitemia is a measure of the number of parasite-infected RBCs relative to the total number of RBCs in a culture. For this EV isolation protocol, however, cultures are diluted with fresh RBCs to a lower hematocrit of 1–2% in large volumes of the culture medium to obtain higher-parasitemia cultures of ≥10% (Appendix A, Table A1). A low hematocrit is also beneficial for malaria EV studies as there are fewer RBCs in the culture while the biomass of parasites in iRBCs (releasing the EV of interest) is simultaneously increased. By alternating sorbitol and Percoll synchronization techniques, high-parasitemia cultures are kept synchronous, i.e., at least 85% of the parasites in a culture are within a 6 h window of the same life stage [58]. The combination of these techniques is important, as sorbitol synchronization alone cannot sustain short-window synchrony of high-parasitemia, high-volume cultures. Sorbitol lyses mature-stage iRBCs, thereby preserving ring-iRBCs in a culture (Figure 4A).

On the other hand, Percoll synchronization is a technique that recovers over 95% of mature-stage iRBCs from culture by separating them from RBCs and early-stage-iRBCs in a density gradient (Figure 4B). Percoll synchronization is ideal when parasites are transferred from old RBCs (~14 days old) to new RBCs, since this technique recovers only mature-stage iRBCs that eventually rupture to release merozoites that invade fresh RBCs. Successfully layering iRBC pellets on a Percoll gradient in the protocol described here may be challenging; however, we provide recommendations on how this can be addressed (Appendix B). The experimental plan and life stage of interest determine the time at which CCM is harvested. This protocol describes the harvesting of CCM from synchronous cultures at 20–22 h, 36–38 h, and 44–46 h post-RBC invasion for isolating EVs from *P. falciparum* ring-, trophozoite-, and schizont-iRBCs, respectively. Radfar et al. provide a detailed pictorial guide of parasite life stages to estimate the time of invasion [58].

**Figure 5:**
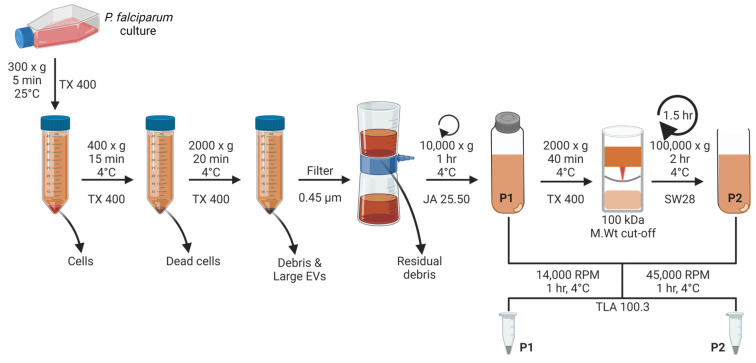
Malaria EV isolation protocol by differential centrifugation. CCM is harvested from a *P. falciparum* culture by centrifugation at 300× *g* for 5 min. The cell pellet is returned to culture and the supernatant is passed through 2 further centrifugations at an increasing speed and time, as well as a filtration step to remove potential contaminants, such as *P. falciparum* merozoites, dead cells, debris, and large EVs. An EV pellet (P1) is isolated from the supernatant at 10,000× *g*. The supernatant can then be concentrated to reduce its volume. A second EV pellet (P2) is isolated from the supernatant at 100,000× *g*. Centrifugation of the pellets in a tabletop ultracentrifuge rotor such as TLA100.3 is highly recommended for the recovery of concentrated EVs.

Two EV preparations of each life stage, each representing a distinct EV subtype, are isolated from a CCM sample using this DC protocol. As is standard for EV isolation by DC, the harvested CCM is first subjected to 2 sequential low-speed centrifugation steps to rid it of *P. falciparum* merozoites, dead cells, large EVs, and debris (Figure 5). We introduced a filtration step to remove remnant debris from the supernatant CCM, and this is followed by medium-speed centrifugation of the supernatant at 10,000× *g* to obtain the first-stage specific EV pellet (P1). In many other protocols, this medium-speed centrifugation pellet is often discarded as debris. However, EVs are pelleted at medium-speed centrifugation [22,63], including budding microvesicles from RBC membranes [46] and malaria-derived EVs [49,64]. With large working volumes of CCM, the supernatant from the medium-speed centrifugation step is concentrated using 100,000 MWCO PES Vivacell units with a centrifugal operation to obtain a more manageable volume for ultracentrifugation. Smaller volumes of CCM are processed directly with no observed effect on EV yield or characteristics. Nevertheless, we recommend that the use of a concentration technique be reported in malaria EV studies (Appendix A, Table A2). The second-stage specific EV pellet (P2) is obtained following high-speed ultracentrifugation at 100,000× *g* in an SW28 rotor. Both pellets (P1 and P2) are concentrated using a TLA100.3 rotor at the equivalent centrifugation speeds at which they were originally pelleted.

Different rotors and (ultra)centrifuges are available to different research groups. Applying this protocol and the centrifugal forces used here on different rotors will likely yield qualitatively and quantitatively different EV preparations. To maximize this protocol, as well as ensure reproducibility and reliable inter-study comparisons, it is essential that the centrifugal forces applied here be transferred to the rotor to be used. Although this can be achieved by manual rotor calculations [38], an excellent and simple resource to perform such calculations is the Intellifuge calculator by Beckman Coulter Life Sciences. This is an online software tool to calculate the centrifugation time, rotor speed, centrifugal force, and K-factor for any combination of tubes, rotors, and centrifuges [65]. With the Intellifuge software, centrifugation protocols can be transferred from one rotor to another, and the configuration of the centrifugation parameters can be easily compared for 2 rotors [65]. We used this online tool to adapt our protocol to isolate the first EV pellet using a different rotor than what is represented in Figure 5 and described in the procedure section (Appendix C).

Generally, EV preparations are analyzed by a combination of techniques, including BCA protein assay, Western blot analysis (WBA), flow cytometry (FC), high-resolution imaging techniques (such as transmission electron microscopy, TEM), and nanoparticle tracking analysis (NTA). The BCA protein assay is useful to determine the protein concentration of EVs, and EVs are analyzed for specific proteins by techniques such as WBA, FC, and various imaging techniques. Microscopic imaging is highly useful for visualizing single EVs, and the concentration of EV preparations can be determined by NTA. Detailed here is a WBA protocol for characterizing malaria-derived EVs. RBC EV markers and membrane proteins, including glycophorin A (GPA), band 3, flotillin 1, flotillin 2, and stomatin, can be analyzed by WBA. Parasite proteins can also be analyzed. RBC ghosts and cytosol from healthy RBCs, as well as parasite lysates, serve as controls for WBA. Although this protocol does not describe EV analysis by NTA, TEM, and FC, representative data from these analyses of malaria-derived EVs isolated using this protocol are presented in Appendix D.

### 2.1. Materials

1. AlbuMAX^TM^ I Lipid-rich BSA (Thermo Fisher Scientific, Saint-Laurent, QC, Canada; Cat. no.: 11020-039)
2. Anti-band 3 (Sigma Life Science, Darmstadt, Germany; Cat. no.: B9277)
3. Anti-PfEBA-175 (BEI Resources, Manassas, VA, USA; Cat. no.: MRA-711A)
4. Anti-PfGRP78 (BEI Resources, Manassas, VA, USA; Cat. no.: MRA-1246)
5. Anti-flotillin 2 (Abcam, Toronto, ON, Canada; Cat. no.: ab181988)
6. Anti-glycophorin A (Abcam, Toronto, ON, Canada; Cat. no.: ab129024)
7. Anti-PfHAP (BEI Resources, Manassas, VA, USA; Cat. no.: MRA-811A)
8. Anti-hemoglobin (Thermo Fisher Scientific, Rockford, IL, USA; Cat. no.: PA5-97488)
9. Anti-mouse secondary antibody (Abcam, Toronto, ON, Canada; Cat. no.: ab6789)
10. Anti-rabbit secondary antibody (Abcam, Toronto, ON, Canada; Cat. no.: ab97080)
11. Bottle-top filter, 0.1 µm (VWR, Radnor, PA, USA; Cat. no.: 89220-696)
12. Bottle-top filter, 0.45 µm (Fisherbrand, Pittsburgh, PA, USA; Cat. no.: FB12566511)
13. Bromophenol blue (Bioshop, Burlington, ON, Canada; Cat. no.: BR0222.10)
14. Chemiluminiscent substrate, Supersignal West Pico PLUS (Thermo Fisher Scientific, Waltham, MA, USA; Cat. no.: A43840)
15. D-Glucose (Bioshop, Burlington, ON, Canada; Cat. no.: GLU501.500)
16. DPBS 1× (Life Technologies Limited, Paisley, UK; Cat. no.: 14190-144)
17. D-Sorbitol (Fisher Chemical, Fair Lawn, NJ, USA; Cat. no.: S459-500)
18. DTT (Bioshop, Burlington, ON, Canada; Cat. no.: DTT001.5)
19. Falcon centrifuge tubes, 15 mL (Corning Science, Reynosa, Tamaulipas, Mexico; Cat. no.: 352099)
20. Falcon centrifuge tubes, 50 mL (Corning Science, Reynosa, Tamaulipas, Mexico; Cat. no.: 352070)
21. Filter paper (Bio Basic, Markham, ON, Canada; Cat. no.: PP3324)
22. Filtered pipette tips, 200 µL (VWR, Radnor, PA, USA; Cat. no.: 76322-150)
23. Filtered pipette tips, 1250 µL (VWR, Radnor, PA, USA; Cat. no.: 76322-156)
24. Gentamicin Reagent Solution 50 mg/mL (Thermo Fisher Scientific, Grand Island, NY, USA; Cat. no.: 15750-060)
25. Giemsa Stain, Modified solution (Sigma-Aldrich, St. Louis, MO, USA; Cat. no.: 48900-1L-F)
26. Glass Pasteur pipettes (Fisher Scientific, Waltham, MA, USA; Cat. no.: 13-678-20C)
27. Glass slides (Azer Scientific, Morgantown, PA, USA; Cat. no.: EMS200W)
28. Glycerolyte^TM^ 57 Solution (Fenwal, Inc., Lake Zurich, IL, USA: Cat no.: 4A7831).
29. Glycine (Bioshop, Burlington, ON, Canada; Cat. no.: GLN001.1)
30. Hypoxanthine (Sigma-Aldrich, St. Louis, MO, USA; Cat. no.: H9377)
31. Immersion oil, type B (Cargille Laboratories, Cedar Grove, NJ, USA; Cat. no.: 16484)
32. KCl (Bioshop, Burlington, ON, Canada; Cat. no.: POC888.1)
33. KH_2_PO_4_ (Bioshop, Burlington, ON, Canada; Cat. no.: PPM302.500)
34. Methanol (Fisher Chemical, Fair Lawn, NJ, USA; Cat. no.: A412)
35. Microcentrifuge tubes, 0.6 mL (Fisher Scientific, Waltham, MA, USA; Cat. no.: 05-407-16)
36. Microcentrifuge tubes, 1.5 mL (Fisher Scientific, Waltham, MA, USA; Cat. no.: 05-408-129)
37. Microfuge tubes, 1.5 mL, polypropylene (Beckman Coulter, Brea, CA, USA; Cat. no.: 357448)
38. Microplate, flat-bottom, 96-well (Fisher Scientific, Waltham, MA, USA; Cat. no.: 12565501)
39. NaCl (Bioshop, Burlington, ON, Canada; Cat. no.: SOD004.5)
40. Nalgene^TM^ Oak-Ridge high-speed polycarbonate centrifuge tubes, 50 mL (Thermo Scientific, Waltham, MA, USA; Cat. no.: 3118-0050PK)
41. NaOH (Fisher Chemical, Fair Lawn, NJ, USA; Cat. no.: S318-500)
42. Na_2_HPO_4_ (Bioshop, Burlington, ON, Canada; Cat. no.: SPD579.1)
43. NaH_2_PO_4_ (Bioshop, Burlington, ON, Canada; Cat. no.: SPM400.1)
44. Needles, 16-gauge (Sigma-Aldrich, St. Louis, MO, USA; Cat. no.: Z118036-100EA)
45. No-stain protein labelling reagent (Thermo Fisher Scientific, Carlsbad, CA, USA; Cat. no.: A44449)
46. Open-top thickwall polypropylene tubes, 32 mL (Beckman Coulter Life Sciences, Indianapolis, IN, USA; Cat. no.: 355642)
47. Percoll^TM^ PLUS (Cytiva Sweden AB, Uppsala, Sweden; Cat. no.: 17544501)
48. Pierce^TM^ BCA protein assay kit (Thermo Fisher Scientific, Rockford, IL, USA, Cat. no.: 23227)
49. Pierce^TM^ Silver stain kit (Thermo Fisher Scientific, Rockford, IL, USA, Cat. no.: 24612)
50. *Plasmodium falciparum* 3D7 strain (BEI Resources, Manassas, VA, USA; Cat. no.: MRA-102)
51. Pre-cast gels, tris-glycine, 10–20%, (Thermo Fisher Scientific, Carlsbad, CA, USA; Cat. no.: XP10205BOX)
52. Pre-cast gels, tris-glycine, 4–12%, (Thermo Fisher Scientific, Carlsbad, CA, USA; Cat. no.: XP04120BOX)
53. Protease inhibitor (Thermo Fisher Scientific, Rockford, IL, USA, Cat. no.: 78430)
54. Protein ladder, Pageruler^TM^ Plus (Thermo Fisher Scientific, Saint-Laurent, QC, Canada; Cat. no.: 26619)
55. PVDF membrane (Millipore, Tullagreen, Carrigtwohill, Co., Cork, Ireland; Cat. no.: IPVH00010)
56. RIPA lysis buffer (Thermo Fisher Scientific, Saint-Laurent, QC, Canada; Cat. no.: 89900)
57. RPMI 1640, 1× (Wisent Inc., Saint-Jean-Baptiste, QC, Canada; Cat. no.: 350-005-CL)
58. Saponin (Sigma-Aldrich, St. Louis, MO, USA; Cat. no.: S4521-10G)
59. SDS (Bioshop, Burlington, ON, Canada; Cat. no.: SDS001.1)
60. Serological pipettes, 1 mL (Thermo Scientific, Saint-Laurent, QC, Canada; Cat. no.: 170353N)
61. Serological pipettes, 5 mL (Fisherbrand, Pittsburgh, PA, USA; Cat. no.: 13-678-11D)
62. Serological pipettes, 10 mL (Fisherbrand, Pittsburgh, PA, USA; Cat. no.: 13-678-11E)
63. Serological pipettes, 25 mL (Fisherbrand, Pittsburgh, PA, USA; Cat. no.: 13-676-10K)
64. Skim milk powder (Bioshop, Burlington, ON, Canada; Cat. no.: SKI400.1)
65. SuperSignal^TM^ West Pico PLUS chemiluminiscent substrate (Life Technologies, Carlsbad, CA, USA; Cat no.: 34580)
66. Syringes, 1 mL (BD, Franklin Lakes, NJ, USA, Cat. no.: 309628)
67. Tissue culture flasks; 250 mL, sterile, non-treated with vent cap (VWR, Radnor, PA, USA; Cat. no.: 10861-576)
68. Tris (Bioshop, Burlington, ON, Canada; Cat. no.: TRS001.5)
69. TX-400 rotor adapters (Thermo Fisher Scientific, Waltham, MA, USA; Cat. no.: 75003788).
70. Tween 20 (ACP Chemicals, Saint-Leonard, QC, Canada; Cat. no.: T-9783)

### 2.2. Equipment

1. Biological safety cabinet, 1300 Series Class II (Thermo Fisher Scientific, Marietta, OH, USA; Cat. no.: 1385)
2. Centrifuge rotor, JA-25.50 fixed angle rotor, 8 × 50 mL, 25,000 rpm (Beckman Coulter Life Sciences, Indianapolis, IN, USA; Cat. no.: 363055)
3. Centrifuge rotor, SW-28 Swinging bucket rotor, 6 × 32 mL, 28,000 rpm (Beckman Coulter Life Sciences, Indianapolis, IN, USA; Cat. no.: 342207)
4. Centrifuge rotor, TLA-100.3 fixed angle rotor, 6 × 4 mL, 100,000 rpm (Beckman Coulter Life Sciences, Indianapolis, IN, USA; Cat. no.: 349490)
5. Centrifuge, Avanti J-E (Beckman Coulter, Palo Alto, CA, USA; Cat. no.: 369001)
6. Centrifuge, Optima Ultracentrifuge L-100 XP (Beckman Coulter, Palo Alto, CA, USA; Cat. no.: 392052)
7. Centrifuge, Optima Ultracentrifuge MAX-130K (Beckman Coulter, Palo Alto, CA, USA; Cat. no.: 364301)
8. Centrifuge, Sorvall with TX400 rotor (Thermo Scientific Waltham, MA, USA; Cat. no.: 75004381)
9. Electronic pipette filler (Corning Science, Glendale, AZ, USA; Cat. no.: 4099)
10. Heat block (Eppendorf, Hamburg, Germany; FisherSci Cat. no.: 05-400-205)
11. Imaging system, Chemidoc MP (Bio-Rad, Saint-Laurent, QC, Canada; Cat. no.: 12003154)
12. Incubator, Heracell 240i (Thermo Scientific, Waltham, MA, USA; Cat. no.: 51032875)
13. Light microscope, Axio Scope.A1 (Carl Zeiss, Gottingen, Germany; Cat. no.: 430035-9040-000)
14. Manual differential counter (Fisherbrand, Saint-Laurent, Quebec, Canada; Cat. no.: 13-684-141)
15. Micropipette, 20–200 µL (Gilson, Middleton, WI, USA; Cat. no.: FA10005M)
16. Micropipette, 100–1000 µL (Gilson, Middleton, WI, USA; Cat. no.: FA10006M)
17. Milli-Q^®^ IQ 7000 ultrapure water system (Sigma-Aldrich, St. Louis, MO, USA; Cat. no.: ZIQ7000T0C)
18. Mini blot module (Thermo Fisher Scientific, Saint-Laurent, QC, Canada; Cat. no.: B1000)
19. Mini centrifuge (Fisher Scientific, Waltham, MA, USA; Cat. no.: 05-090-100)
20. Mini gel tank (Thermo Fisher Scientific, Saint-Laurent, QC, Canada; Cat. no.: A25977)
21. Multichannel micropipette (Sartorius, Gottingen, Germany; Fisher Scientific Cat. no.: 720240)
22. Power supply (Bio-Rad, Saint-Laurent, QC, Canada; Cat. no.: 1645052)
23. Plater reader (BioTek Instruments, Winooski, VT, USA; Product ID: Synergy H4)
24. Rocker (Boekel Scientific, Feasterville-Trevose, PA, USA; Cat. no.: 260350)
25. Vacuum pump (Welch, Monroe, LA, USA, Cat. no.: 2534B-01)
26. Vivacell^®^ 100, 100,000 MWCO PES (Sartorius, Stonehouse, UK; Cat. no.: VC1042)
27. Vortex mixer (Fisher Scientific, Waltham, MA, USA; Cat. no.: 02215365)

## 3. Procedure

### 3.1. Preparing and Maintaining P. falciparum Cell Culture for EV Isolation (Figure 3, Top)


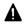
 **CRITICAL STEP.** Conduct all steps with asceptic techniques, performed under a class 2 biological safety cabinet. Wear personal protective equipment at all times.

#### 3.1.1. Preparation of Fresh RBCs: Time to Complete: ~1 h

1. Aliquot ~30 mL of fresh leukoreduced RBCs into sterile 50 mL tubes.
2. Using any suitable tabletop centrifuge, spin at 2000 rpm for 10 min at 4 °C. Discard supernatant.

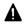
 **CRITICAL STEP.** Completely remove the top ~1 mL layer of RBCs. This is extremely important if using non-leukoreduced RBCs. For optimal *P. falciparum* cultures intended for EV isolation from CCM, leukoreduced blood is preferred. If leukoreduced blood is not available, carefully wash RBCs to minimize buffy coat remnants, which contain white blood cells and platelets after centrifugation. For downstream experiments on isolated EVs, evaluate the presence of non-RBC-derived EV markers, such as CD63 and CD9, using Western blot analysis or flow cytometry. Interpret all findings carefully, considering the potential for contamination.

3. Resuspend RBCs in an equal volume of incomplete medium and centrifuge at 2000 rpm for 10 min at 4 °C. Discard supernatant.
4. Repeat wash step 3.
5. Dilute the washed RBCs to 50% hematocrit by resuspending in an equal volume of incomplete medium.
6. Store the washed RBCs at 4 °C for a maximum of 14 days from the time of collection from donor.

#### 3.1.2. Thawing Parasites Previously Frozen in Glycerol: Time to Complete: ~1 h


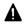
 **CRITICAL STEP.** For high-parasitemia cultures to be obtained within a few days of thawing parasites, freeze young synchronous ring cultures at ≥10% parasitemia. Typically, store 500 µL of packed parasite-infected RBCs in 500 µL glycerol freezing solution (see Section 2.1, Materials).

1. Remove a cryovial of malaria parasites from liquid nitrogen and thaw for 1–2 min at room temperature.
2. Using a 1 mL pipette, transfer the contents of the cryovial to a 15 mL tube.
3. For every 1 mL of thawed parasites (i.e., 500 µL of packed parasite-infected RBCs + 500 µL glycerol freezing solution), slowly add 100 µL of solution I (see Section 5, Reagent Setup) dropwise, shaking gently at intervals to mix the suspension.
4. Incubate the suspension at room temperature for 5 min.
5. For every 1 mL of thawed parasites, slowly add 10 mL of solution II (see Section 5, Reagent Setup) dropwise, shaking gently at intervals to mix the suspension.
6. Centrifuge at 2000 rpm for 5 min at room temperature. Discard the supernatant.
7. **OPTIONAL STEP.** For every 1 mL of thawed parasites, slowly add 10 mL of solution III dropwise, shaking gently at intervals to mix the suspension. Incubate at room temperature for 5 min. Centrifuge at 2000 rpm for 5 min at room temperature. Discard the supernatant.
8. Resuspend the pelleted cells in 10 mL of incomplete medium.
9. Centrifuge at 2000 rpm for 5 min at room temperature. Discard the supernatant.
10. Using a 5 mL pipette, estimate the volume of the pelleted cells by resuspending the pellet in exactly 5 mL of complete medium and determining the extra volume in the pipette.
11. Transfer the cells to a culture flask.
12. Add an equal amount of fresh RBCs as the estimated malaria parasite pellet.

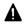
 **CRITICAL STEP.** Add double the volume of required fresh RBCs from the stored 50% hematorit RBCs. For example, 300 µL of packed RBCs are contained in 600 µL of 50% hematorit fresh RBCs.

13. Add the remaining volume of complete medium to obtain a hematocrit of 1%. For example, if 300 µL of iRBC pellet was recovered after thawing, the final composition of the culture with 1% hematocrit will be

300 µL iRBC + 5 mL complete medium + 300 µL packed fresh RBCs + 55 mL complete medium

14. Incubate the culture flask in 5% CO_2_, 3% O_2_, and 95% N_2_ at 37 °C for 48 h.

#### 3.1.3. Culture Check and Culture Medium Changes: Time to Complete: ~30 min


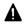
 **CRITICAL STEP.** Always examine cultures by a thin Giemsa-stained smear to determine the parasite’s life stage, viability, and parasitemia prior to processing and changing the growth medium.

1. After 48 h incubation, use a 1 mL pipette to transfer 100 µL of the culture into a 0.6 mL microcentrifuge tube.
2. Centrifuge for 5–10 s in a mini centrifuge.
3. On a clean glass slide, make a thin smear using 2 µL of the pelleted cells. Allow to air-dry completely.
4. Fix the smear by dipping in methanol for 3–5 s. Allow to air-dry completely.
5. Prepare a 10% Giemsa solution with distilled water in a slide staining jar.
6. Completely immerse the smear in the 10% Giemsa solution and leave to stain for 8–10 min.
7. Rinse the slide thoroughly with water. Allow to air-dry completely.
8. Examine under the light microscope using the 100× immersion oil objective lens. Parasites should be at the ring stage.
9. Properly resuspend cells in the flask and transfer culture to a 50 mL tube.
10. Centrifuge at 2000 rpm for 5 min at room temperature. Discard the supernatant.
11. Resuspend cells in the appropriate volume of fresh culture medium to maintain a 1% hematocrit and return to the flask. Since the culture is expected to contain ring stages, do not add RBCs.
12. Reincubate the flask for 24 h in 5% CO_2_, 3% O_2_, and 95% N_2_ at 37 °C.
13. After 24 h, prepare, stain, and examine a smear of the culture as described in steps 1–8 above. Parasites should be late trophozoites or early schizonts.
14. Determine the percentage parasitemia and estimate the time of invasion. Note: This will vary slightly depending on the parasite strain used.
15. Estimate the volume of pelleted cells as described in Section 3.1.2, step 10.
16. Return cells to the flask. If the parasitemia is <10%, proceed to step 17. If the parasitemia is ≥10%, proceed to step 18.
17. If the parasitemia is <10%, resuspend the pelleted cells in at least 100 times its volume of fresh culture medium. For example, a 600 µL cell pellet will be resuspended in 60 mL of fresh culture medium. Do not add RBCs. Proceed to step 19.

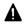
 **CRITICAL STEP.** Earlier protocols for high-parasitemia cultures of *P. falciparum* have established that 2.5 mL of a culture medium is required to support the growth of ring stages in a 50 µL packed cell culture at 10% parasitemia [66]. By doubling this volume when parasites are at the mature stages, as well as not adding RBCs, the parasitemia will be significantly increased at the next invasion with enough culture medium available to sustain the parasitemia.

18. If the parasitemia is ≥10%, add 1 volume of packed fresh RBCs to 3 volumes of pelleted cell culture and resuspend the cells in sufficient fresh culture medium to restore the 1% hematocrit. For example, add 200 µL of packed fresh RBCs to 600 µL of pelleted iRBC culture in 80 mL of fresh culture medium.
19. Reincubate the flask in 5% CO_2_, 3% O_2_, and 95% N_2_ at 37 °C until sorbitol synchronization.

#### 3.1.4. Sorbitol Synchronization: Time to Complete: ~40 min

1. After 12–16 h of incubation, or ~6 h after the time of invasion estimated in Section 3.1.3, step 14, examine a stained smear of the culture as described in steps 1–8 of Section 3.1.3. If young rings are observed, proceed with sorbitol synchronization; otherwise, recheck cultures after 2–3 h.

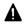
 **CRITICAL STEP.** Perform sorbitol synchronization on cultures of young rings at 6–10 h post-invasion with a parasitemia of ≥10%.

2. Properly resuspend cells in the flask and transfer culture to a 50 mL tube.
3. Centrifuge at 2000 rpm for 5 min at room temperature. Discard the supernatant.
4. Resuspend 1 volume of the pelleted cells in 10 volumes of 5% sorbitol.
5. Vigorously vortex the suspension for 30 s to lyse the mature-stage iRBCs.
6. Incubate for 10 min at room temperature, mixing the suspension by inverting the tube at intervals.
7. Vortex the suspension again for 10 s.
8. Centrifuge at 2000 rpm for 5 min at room temperature. Discard the supernatant.
9. Resuspend the pellet in 10 mL of incomplete medium and centrifuge at 2000 rpm for 5 min at room temperature. Discard the supernatant.
10. Repeat wash step 9.
11. Resuspend the pelleted cells in the appropriate volume of fresh culture medium.

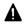
 **CRITICAL STEP.** Determine the volume of fresh culture medium required to sustain a high-parasitemia culture of ring-iRBCs for at least 24 h using the following formula [58]:

Volume of fresh medium (mL)/24 h = 0.005 × (µL RBC pellet) × (% parasitemia)

12. Transfer the culture to a new culture flask.

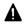
 **CRITICAL STEP.** Do not return the culture to the old flask, as the presence of residual mature-stage parasites will disrupt the synchronization window.

13. Reincubate the flask in 5% CO_2_, 3% O_2_, and 95% N_2_ at 37 °C until the next media change.
14. Over the next 48 h of incubation, check the culture and perform media changes as described in Section 3.1.3. Add fresh RBCs when parasites are at mature stages. Estimate the time of invasion to determine when the parasites will be ready for Percoll synchronization.

#### 3.1.5. Percoll Synchronization—Adapted from [67]: Time to Complete: ~1 h 30 min

1. After ~40 h post invasion (as determined by the time of invasion estimated in the previous step), examine a stained smear of the culture. If schizonts are mainly observed, proceed with Percoll synchronization.

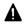
 **CRITICAL STEP.** Perform Percoll synchronization on cultures of mature schizonts at ≥40 h post-invasion with a parasitemia of ≥10%.

2. In a 15 mL tube, add 3 mL 65% Percoll solution using a 5 mL pipette.
3. Layer 3 mL of 35% Percoll solution on top of the 65% Percoll solution using a 16-gauge needle and 1 mL syringe.

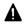
 **CRITICAL STEP.** Layer the gradient very carefully to form a clear interphase between both Percoll concentrations. Lean the needle against the wall of the tube with the bore visible just above the 65% Percoll layer. Release the 35% Percoll solution dropwise while slowly rotating the tube to allow for even distribution. Using a needle to layer the gradient offers more control over using a pipette due to the smaller bore size.

4. Properly resuspend cells in the flask and transfer the culture to a 50 mL tube.
5. Centrifuge at 2000 rpm for 5 min at room temperature. Discard the supernatant.
6. Layer the cells on top of the Percoll gradient using a 16-gauge needle and 1 mL syringe.

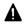
 **CRITICAL STEP.** Layer the cells quickly but carefully so as not to disturb the interface between the cells and 35% Percoll solution. Use the same technique described above. If this cannot be achieved, see Appendix B.

7. Immediately centrifuge at 2500 rpm for 15 min at room temperature with medium acceleration and no brake to preserve the gradient.
8. Carefully remove the top layer, first interface (culture medium, dead cells, and debris), and 35% Percoll layer with a 5 mL pipette (Figure 4B).
9. Carefully transfer the interface that contains the mature-stage iRBCs to a 15 mL tube using a glass Pasteur pipette and pipette bulb. Discard the 65% Percoll layer and pellet of RBCs and early-stage iRBCs.
10. Add 10 mL of incomplete medium to the recovered mature-stage iRBCs by slowly releasing it down the wall of the tube. Mix by gentle inversion.
11. Centrifuge at 2000 rpm for 5 min at room temperature. Discard the supernatant.
12. Repeat wash steps 10 and 11.
13. Resuspend the pellet of mature-stage iRBCs in 10 mL of fresh complete culture medium and transfer to a new flask.

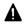
 **CRITICAL STEP.** Do not return the culture to the old flask as the presence of residual younger-stage parasites will disrupt the synchronization window.

14. Add 500 µL of packed fresh RBCs and 40 mL of fresh culture medium to give a final hematocrit of 1%.

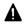
 **CRITICAL STEP.** Estimate the pellet volume of mature-stage iRBCs, as adding too much fresh RBCs will greatly reduce the parasitemia at the next invasion. The volumes suggested here are based on ~100 µL that can be recovered from 1 mL of iRBCs with a schizont parasitemia of ≥10%. When maintaining long-term continuous cultures, Percoll synchronization should be timed to transfer parasites from ≤14-day-old RBCs to fresh RBCs.

15. Incubate the flask in 5% CO_2_, 3% O_2_, and 95% N_2_ at 37 °C.
16. Check after ~10 h for young ring-iRBCs and change the culture medium as described in Section 3.1.3. Calculate the parasitemia and use the formula described in Section 3.1.4, step 11, to determine the volume of fresh culture medium required to sustain the parasites until the next culture check and CCM harvesting.
17. Estimate the time of invasion so that the growth cycle can be followed accurately, and CCM harvesting timed accordingly.

### 3.2. Harvesting EV-Containing CCM from P. falciparum Cultures: Time to Complete: ~2 h (Figure 3, Bottom)


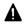
 **CRITICAL STEP.** High parasitemia cultures require extra attention to ensure that cells remain viable, healthy, and free of contamination. Harvest CCM only from cultures that fulfill all these criteria. After 7–8 days of thawing frozen rings (≥10% parasitemia) and performing sorbitol and Percoll synchronization, high-parasitemia, large-volume cultures are ready for harvesting the CCM from the parasite’s life stage of interest for EV studies. Perform culture checks and medium changes, as described in Section 3.1.3. The appropriate volume of culture medium should be determined using the formula in Section 3.1.4, step 11.

1. Examine the culture at 20–22 h post invasion to verify that the parasites are at the late ring stage.
2. Properly resuspend cells in the flask and transfer culture to a 50 mL tube.
3. Centrifuge at 2000 rpm for 5 min at room temperature. Transfer the supernatant to new 50 mL tubes and store at 4 °C for ~30 min, while the cells are prepared for reincubation. The CCM contains EVs from ring-iRBCs.
4. Resuspend the cells in the appropriate volume of culture medium and reincubate the flask in 5% CO_2_, 3% O_2_, and 95% N_2_ at 37 °C until the next culture change or harvesting of trophozoite-iRBCs CCM (steps 5 and 6). Proceed to step 12.
5. Examine the culture at 36–38 h post invasion to verify that the parasites are at the late trophozoite stage.
6. Repeat steps 2 and 3. The CCM contains EVs from trophozoite-iRBCs.
7. Resuspend the cells in the appropriate volume of culture medium and reincubate the flask in 5% CO_2_, 3% O_2_, and 95% N_2_ at 37 °C until the next culture change or harvesting of schizont-iRBCs CCM (steps 8 and 9). Proceed to step 12.
8. Examine the culture at 44–46 h post invasion to verify that the parasites are at the mature schizont stage.
9. Repeat steps 2 and 3. The CCM contains EVs from schizont-iRBCs.
10. Subculture the schizont-iRBCs by dividing the cell pellet evenly into 2 new flasks. Add fresh RBCs and the appropriate volume of complete culture medium to obtain a 1% hematocrit.
11. Reincubate the flasks in 5% CO_2_, 3% O_2_, and 95% N_2_ at 37 °C until the next culture change or harvesting of ring iRBCs CCM. Proceed to step 12.

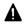
 **CRITICAL STEP.** From this point, CCM is harvested from 2 flasks, each with synchronous, high-parasitemia cultures. Cultures will remain highly synchronized for at least 2 growth cycles, after which sorbitol and Percoll synchronization should be repeated. To continue harvesting CCM, repeat steps 1–11. Although it is possible to subculture the parasites into 4 flasks at step 10 to harvest more CCM, this is a cumbersome task. We do not recommend working with more than 2 culture flasks (each with a maximum of 120 mL of culture) at a time. Excess parasites can be reincubated until the ring stage and then frozen.

12. Retrieve the CCM from the 4 °C storage and centrifuge at 400 g and 4 °C for 15 min in a Sorvall ST 16R centrifuge with a TX 400 rotor. Acceleration 9, deceleration 7. This step clears the CCM of dead cells.
13. Carefully transfer the supernatant to a new 50 mL tube(s) using a 25 mL pipette.

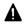
 **CRITICAL STEP.** Leave ~0.5 mL of supernatant over the pellet to ensure that the pellet is not collected. Do not contaminate the supernatant with the pellet.

14. Centrifuge the supernatant at 2000× *g* and 4 °C for 20 min in a Sorvall ST 16R centrifuge with a TX 400 rotor. Acceleration 9, deceleration 7. This step clears the CCM of debris and large EVs.
15. Carefully transfer the supernatant (CCM) to a new 50 mL tube(s) using a 25 mL pipette.

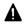
 **CRITICAL STEP.** Leave ~0.5 mL of supernatant over the pellet to ensure that the pellet is not collected. Do not contaminate the supernatant with the pellet.


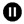
 **PAUSE STEP.** The supernatant from the 2000× *g* spin can be stored at 4 °C for up to 3 days or at −80 °C for 2 months until needed for EV isolation. We do not recommend storing CCM for longer periods of time under either of these conditions, as EVs may degenerate during storage. EVs should be isolated from CCM as soon as possible.

### 3.3. Isolation of Malaria-Derived EVs by Differential Centrifugation (Figure 5)

#### 3.3.1. Isolation of EV Pellet 1 (P1): Time to Complete: 5–6 h

1. If CCM from Section 3.2, step 15, was frozen, thaw it in a refrigerator at 4 °C over 24–48 h.

**CRITICAL STEP.** Do not thaw CCM at room temperature.

2. Pass the CCM through a 0.45 µm PES membrane bottle-top filter to remove any remaining debris.
3. Collect the supernatant and transfer it into 50 mL polycarbonate centrifuge tubes.
4. Mark a dot on one side of each centrifuge tube, close to the bottom, and position the tubes in a JA-25.50 fixed-angle rotor such that the dots are facing up.

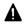
 **CRITICAL STEP.** Fixed-angle rotors cause pellets to sediment on the side of the tube that is furthest away from the rotor’s center of rotation. Marking the bottom side of the tubes makes it easier to locate the near “invisible” EV pellet. Reuse tubes to centrifuge CCM from the same sample (no more than once, to avoid loss of EV integrity) to increase the pellet size and improve visibility.

5. Centrifuge at 10,000× *g* (9000 rpm) and 4 °C for 1 h with maximum acceleration and slow deceleration. Note: The Avanti-J.E centrifuge used here in this protocol has only two options for acceleration and deceleration. These are maximum or slow.
6. Using a 25 mL pipette, carefully collect the supernatant into a sterile glass bottle. Do not disturb the pellet.

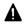
 **CRITICAL STEP.** To remove the supernatant, tilt the tube backward and position the end of the pipette at the opposite side of the pellet. Leave ~0.5 mL of supernatant above the pellet.

7. Store the supernatant at 4 °C until needed for isolation of pellet 2.

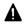
 **CRITICAL STEP.** Do not store the supernatant for more than 24 h.

8. Pool the pellets and remaining supernatant from step 6 into a single 50 mL polycarbonate centrifuge tube.
9. Using 5 mL pipettes, wash all tubes 2–3 times with PBS by carefully flooding the location of the pellet, and pool the PBS washes into the collection tube with the original pellet. Fill the collection tube with PBS.

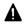
 **CRITICAL STEP.** If pellets will be analyzed by NTA or flow cytometry, pass the PBS through a 0.2 µm filter (preferably 0.1 µm) before using it in any steps of EV isolation. This is necessary to remove any particles present in the PBS that may affect NTA or flow cytometry readings.

10. Centrifuge in a JA-25.50 fixed-angle rotor at 10,000× *g* (9000 rpm) and 4 °C for 1 h with maximum acceleration and slow deceleration to wash the pellet.
11. Remove the supernatant using the same technique described in step 6.
12. Gently tilt the tube towards the opposite side of the pellet and pour off the remaining supernatant. When visible, the pellet appears cloudy-white.
13. Leave the tube upside-down over a Kimwipe for 30 s to 1 min.
14. **OPTIONAL STEP.** Carefully wipe dry the mouth and the top half of the insides of the tube with Kimwipes. Do not touch the pellet.
15. Resuspend the pellet in 1.2 mL PBS and transfer to a 1.5 mL microfuge tube. This step should be carried out by performing 3 washes of the tube, each with 400 µL of PBS, and pooling the pellet + PBS washes in the 1.5 mL microfuge tube.

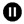
 **PAUSE STEP.** The pellet suspension in microfuge tubes can be stored at 4 °C overnight.

16. Concentrate the EV pellet by centrifuging in a TLA100.3 rotor at 14,000 rpm (10,000 g) and 4 °C for 1 h. Acceleration 9, deceleration 7.

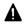
 **CRITICAL STEP.** Position the tube in the rotor with its lid hinge facing up to mark the location of the pellet. Like the JA-25.50 rotor, the TL100.3 is a fixed-angle rotor that causes pellets to sediment on the side of the tube that is furthest away from the rotor’s center of rotation.

17. Remove the supernatant completely using a 1 mL filtered pipette tip. Do not touch the pellet.

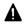
 **CRITICAL STEP.** First remove half of the supernatant and any residual supernatant around the tube cap and mouth. Then, position the pipette tip at the opposite side of the pellet and remove the remaining supernatant. Hold the tube up and wait a few seconds to allow any remnant supernatant to slide down the tube so this can also be removed.

18. Resuspend the pellet in 50–100 µL of PBS and aliquot as necessary to avoid freeze–thaw cycles.
19. Analyze P1 EVs immediately or store at −80 °C for up to 2 months.

#### 3.3.2. Isolation of EV Pellet 2 (P2): Time to Complete: 7–9 h

1. Retrieve the supernatant from Section 3.3.1, step 7, from 4 °C storage.
2. Using a 25 mL pipette, transfer the supernatant to Vivacell 100 units.

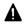
 **CRITICAL STEP.** Vivacell units do not come sterile. Clean Vivacells before and after use according to the manufacturer’s directions.

3. Concentrate the supernatant by centrifuging the Vivacells in a Sorvall ST 16R centrifuge with a TX 400 rotor at 2000× *g* and 4 °C for 30–40 min.

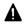
 **CRITICAL STEP.** Use the appropriate TX 400 rotor adapter to accommodate Vivacells (see Section 2.1, Materials).

4. Discard the flow-through.
5. Using a combination of 1 mL and 200 µL filtered pipette tips, carefully transfer the concentrates from the Vivacells to a 15 or 50 mL tube.

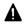
 **CRITICAL STEP.** Tilt the vivacells, allowing the concentrate to flow away from the surface of the membrane. Minimize touching the membrane with the pipette tips to avoid damaging it. We do not recommend using glass Pasteur pipettes to collect the concentrate as this increases the chances of piercing the Vivacell membrane.

6. Repeat step 3 until all of the supernatant has been concentrated. The same Vivacell(s) should be used to process the same sample. Pool concentrates from the same sample into the same collection tube and store at 4 °C until the concentration step is complete.
7. Transfer 5–10 mL of the concentrate to an ultracentrifuge tube. For optimal EV recovery, we do not recommend centrifugation of more than 10 mL of concentrated supernatant per ultracentrifuge tube.
8. Dilute the concentrated CCM in ultracentrifuge tubes appropriately. For example, a concentrate of 5 mL in a 32 mL polypropylene tube should be diluted in 27 mL of PBS.

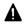
 **CRITICAL STEP.** If pellets will be analyzed by NTA or flow cytometry, pass the PBS through a 0.2 µm filter (preferably 0.1 µm) before using it in any steps of EV isolation. This is necessary to remove any particles present in the PBS that may affect NTA or flow cytometry readings.

9. Centrifuge in an SW-28 swinging bucket rotor at 100,000× *g* (24,000 rpm) and 4 °C for 2 h. Acceleration 3, deceleration 6.
10. Using a 25 mL pipette, carefully remove the supernatant, leaving ~1 mL above the pellet. Fill the tube with PBS. The pellet sediments at the bottom of the tube for swinging bucket rotors.
11. **OPTIONAL STEP.** If more than 1 tube was used for a sample, pool the pellets and remaining supernatant into a single ultracentrifuge tube. Using 5 mL pipettes, wash all tubes 2–3 times with PBS by carefully flooding the bottom of the tube. Pool the washes into the collection tube with the original pellet. Fill the collection tube with PBS.
12. Centrifuge in an SW-28 swinging bucket rotor at 100,000× *g* (24,000 rpm) and 4 °C for 1.5 h to wash the EV pellet. Acceleration 3, deceleration 6.
13. Using a 25 mL pipette, carefully remove the supernatant, leaving ~1 mL above the pellet.
14. Gently tilt the tube and pour off the remaining supernatant. When visible, the pellet appears cloudy-white with a red tint.
15. Leave the tube upside-down over a Kimwipe for 30 s to 1 min.
16. **OPTIONAL STEP.** Carefully wipe dry the mouth and the top half of the sides of the tube with Kimwipes. Do not touch the pellet.
17. Resuspend the pellet in 1.2 mL of PBS and transfer to a 1.5 mL microfuge tube. See Section 3.3.1, step 15, for instructions.
18. Concentrate the EV pellet by centrifuging in a TLA100.3 rotor at 45,000 rpm (100,000× *g*) and 4 °C for 1 h. Acceleration 9, deceleration 7.

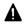
 **CRITICAL STEP.** See Section 3.3.1, step 16, for instructions.

19. Remove the supernatant completely using a 1 mL filtered pipette tip. Do not touch the pellet.

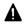
 **CRITICAL STEP.** See Section 3.3.1, step 17, for instructions.

20. Resuspend the pellet in 50–100 µL PBS and aliquot as necessary to avoid freeze–thaw cycles.
21. Analyze P2 EVs immediately or store at −80 °C for up to 2 months.

### 3.4. Analysis of Malaria-Derived EVs by Western Blotting

#### 3.4.1. Preparation of Parasites (WBA Controls) from iRBCs by Saponin Lysis. Adapted from [68]: Time to Complete: ~1 h

1. **OPTIONAL STEP.** Add 5 mM of protease inhibitor to PBS.
2. Add 20 mL PBS to a 50 mL tube.
3. Transfer 50–100 µL of packed cell pellets (1–2 × 10^8^ cells) of ring-, trophozoite-, or schizont-iRBCs (from steps 4, 7, and 10, respectively, of Section 3.2.) to the 50 mL tube. Mix gently.
4. Centrifuge at 800× *g* and 4 °C for 3 min.
5. Remove the supernatant, resuspend the cell pellet in 20 mL of PBS, and centrifuge at 800× *g* and 4 °C for 3 min.
6. Resuspend the washed cell pellet in 50 mL of PBS.
7. Lyse the RBCs to release intact parasites by adding 50 µL of 10% saponin solution.
8. Incubate the tube on ice for 3–5 min, inverting the tube intermittently. During this time, the suspension gradually changes from turbid red to clear red.
9. Centrifuge at 1800× *g* and 4 °C for 3 min.
10. Remove the supernatant that contains the RBC cytosol and parasitophorous vacuolar membranes of the parasites.
11. Resuspend the pellet of intact parasites in 20 mL PBS.
12. Centrifuge at 1800× *g* and 4 °C for 3 min. Remove the supernatant.
13. Resuspend the parasite pellet in 20–50 µL PBS.
14. Aliquot the parasite suspension into microcentrifuge tubes
15. Store at −80 °C until needed.

#### 3.4.2. Preparation of RBC Ghost Membranes and Cytosol (WBA Controls)—Adapted from [69]: Time to Complete: ~2 h

1. Transfer 2 mL of 50% Hematocrit RBCs to a 50 mL tube.

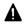
 **CRITICAL STEP.** If a culture of uninfected RBCs has been set up as a control, collect RBCs from this culture to prepare ghost membranes and cytosol.

2. Resuspend the RBCs in 20 mL PBS.
3. Centrifuge at 2000× *g* and 4 °C for 10 min.
4. Remove the supernatant and repeat steps 2 and 3.
5. Add 40 mL of 5 mM sodium phosphate buffer to the washed RBC pellet (now 1 mL).
6. Vortex thoroughly for 30 s to lyse RBCs.
7. Transfer the suspension to a high-speed polycarbonate centrifuge tube.
8. Centrifuge at 20,000× *g* and 4 °C for 15 min with slow deceleration.
9. Carefully collect 100 µL of the supernatant (contains the RBC cytosol) into an Eppendorf tube. Set aside for storage at −80 °C.
10. Carefully remove the remaining supernatant using a 25 mL pipette, leaving ~2 mL over the RBC ghost membranes.

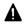
 **CRITICAL STEP.** The RBC ghost membrane pellet is not tightly packed. Care must be taken to not collect it with the supernatant.

11. Slide the loose pellet of RBC ghosts from where it has sedimented by gently tilting and rotating the tube. This gives access to a smaller red pellet of proteases. Note: When lysed RBCs are centrifuged, two pellets are obtained—a larger, loose, and cloudy pellet of RBC ghost membranes, over a smaller, hard, red pellet of proteases.
12. Aspirate the pellet of proteases.
13. Resuspend the RBC ghost membranes in 30 mL of 5 mM sodium phosphate buffer.
14. Centrifuge at 20,000× *g* and 4 °C for 15 min with slow deceleration.
15. Repeat steps 13 and 14.
16. **OPTIONAL STEP.** Add 5 mM of protease inhibitor to the final wash.
17. Aliquot the RBC ghost membranes into Eppendorf tubes.
18. Store at −80 °C until needed.

#### 3.4.3. Preparation of EV and Cell Lysates: Time to Complete: ~1 h

1. Add protease inhibitor to RIPA lysis buffer immediately before use at a concentration of 10 µL/mL.
2. Add 100 µL of the RIPA lysis buffer/protease inhibitor mix to each EV pellet.
3. Add 100 µL of the RIPA lysis buffer/protease inhibitor mix to aliquots of the controls (parasite cell lysates, RBC ghost membrane lysate, and cytosol—see Section 3.4.1 and Section 3.4.2).
4. Vortex the mixture briefly.
5. Incubate on ice for 15–20 min.
6. Vortex the mixture again.
7. Centrifuge the mixture at 14,000× *g* and 4 °C for 15 min to pellet the debris.
8. Transfer the supernatant to new tubes.
9. Store at −20 °C until needed or quantify proteins in the lysates immediately using the microplate procedure of the Pierce^TM^ BCA protein assay, according to the manufacturer’s instructions (see Section 2.1, Materials).

#### 3.4.4. SDS–Polyacrylamide Gel Electrophoresis: Time to Complete: 1.5–2.5 h


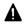
 **CRITICAL STEP.** Set up the electrophoresis system and gels according to the manufacturer’s instructions. This protocol describes the set up for the mini gel tank and pre-cast gels from Thermo Fisher Scientific (see Section 2.1, Materials).

1. Fill the chamber of an electrophoresis gel tank with 1× SDS running buffer.
2. Prepare a pre-cast tris-glycine gel and clamp it in place in the electrophoresis tank.

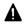
 **CRITICAL STEP.** When using pre-cast gels, choose the right gel format for the experiment, with careful consideration of the gel type, polyacrylamide percentage, molecular weight separation range, and well format.

3. In microcentrifuge tubes, add 6× Laemmli’s buffer to lysates of each isolated EV sample and controls (from Section 3.4.3.) to a final concentration of 1×.
4. Vortex samples and spin down briefly in a mini centrifuge.
5. Incubate the samples in a heat block at 95 °C for 5 min.
6. Vortex samples and spin down briefly in a mini centrifuge.
7. Load 3–5 µL of ladder into the first well.
8. Load 1 µg of protein for each sample and control into remaining wells.
9. Run the gel at 225 V for 20–40 min or until the ladder or dye (from Laemmli’s buffer) almost reaches the foot of the gel.
10. **OPTIONAL STEP.** Perform SDS-PAGE on a separate gel to visualize proteins on the gel by a suitable staining technique, such as silver staining.

#### 3.4.5. Western Blot Analysis (WBA): Time to Complete: 2 Days


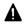
 **CRITICAL STEP.** Set up the protein transfer system and perform all steps of the protein transfer according to the manufacturer’s instructions. This protocol describes the set up for the mini blot module (semi-wet transfer) from Thermo Fisher Scientific (see Section 2.1, Materials).

1. In a membrane tray, activate a PVDF transfer membrane in methanol for 30 s.

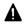
 **CRITICAL STEP.** Only handle the membrane with clean, plastic tweezers to avoid contamination and blotching.

2. Rinse the membrane briefly in deionized water.
3. Equilibrate the PVDF membrane in 1× transfer buffer for at least 5 min.
4. Soak 2 sponge pads thoroughly in transfer buffer.
5. When electrophoresis is complete (Section 3.4.4.), remove the gel cassette from the tank and rinse with deionized water.
6. Using a gel knife, carefully open the gel cassette and trim off the gel wells. The gel remains attached to the long plate of the gel cassette.
7. Wet a filter paper in 1× transfer buffer and apply it to the gel, positioning it above the gel foot.
8. Roll out air bubbles.
9. Carefully detach the gel and filter paper from the cassette plate onto a hard flat-surface, filter paper first.
10. Wet the cathode core with 5–10 mL of 1× transfer buffer.
11. Proceed with assembling the transfer sandwich from bottom to top as follows: cathode core–sponge pad–filter paper–gel–PVDF membrane–filter paper–sponge pad–anode core.

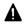
 **CRITICAL STEP.** Use a blotting roller to carefully roll out bubbles at each layer. Air bubbles in the sandwich will impede transfer of the sample proteins to the membrane, resulting in blank patches. Before applying filter paper to the sandwich, soak each piece briefly in 1× transfer buffer.

12. Place the assembled blot sandwich in the electrophoresis tank, ensuring that all inner components are submerged in 1× transfer buffer.
13. Run the transfer at 20 V for 60 min.
14. While the transfer is running, prepare the blocking buffer, incubation buffer, and wash buffer (see Section 5, Reagent setup).
15. When the transfer run is complete, carefully unpack the sandwich and transfer the blotted membrane to a membrane tray with enough deionized water (~20 mL) to completely cover the membrane.
16. **OPTIONAL STEP.** Use the No-Stain^TM^ Protein Labelling Reagent (see Section 2.1, Materials) or any other suitable membrane stain to visualize proteins (and perform normalization for quantitative WBA) on the membrane according to the manufacturer’s instructions.
17. On a shaker, wash the membrane in the deionized water for 5 min. Repeat wash.
18. Block the membrane in 5% TBSM-T blocking buffer on a shaker for 1 h.
19. Completely pour off the blocking buffer and add a primary antibody appropriately diluted in 10 mL of 0.5% TBSM-T incubation buffer.
20. Incubate overnight on a shaker in a cold room at 4 °C.
21. Pour off the primary antibody and rinse the membrane briefly with 0.1% TBS-T.
22. Wash the membrane with 0.1% TBS-T for 10 min on a shaker.
23. Repeat wash step 22 two more times.
24. Add a secondary antibody appropriately diluted in 10 mL of 0.5% TBSM-T incubation buffer. This protocol uses a dilution of 1 in 5000.
25. Incubate for 1 h on a shaker at room temperature.
26. Pour off the secondary antibody and rinse the membrane briefly with 0.1% TBS-T.
27. Wash the membrane with 0.1% TBS-T for 10 min on a shaker.
28. Repeat wash step 27 two more times. Leave the membrane in the last wash solution.
29. Prepare 10 mL of enhanced chemiluminescent (ECL) substrate working solution from an ECL Kit (5 mL of solution A and 5 mL of solution B; see Section 2.1, Materials).
30. Pour off the last wash solution from step 28 and cover the membrane with the ECL substrate working solution.
31. Incubate for 5 min.
32. Transfer the membrane between 2 acetate sheets. Blot any excess ECL solution and remove air bubbles in the membrane–acetate sheet sandwich.

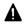
 **CRITICAL STEP.** Place the blotted membrane between acetate sheets to prevent it from drying out during imaging.

33. Image the membrane in a suitable imaging system (see Section 2.2, Equipment).

## 4. Expected Results

**Figure 6:**
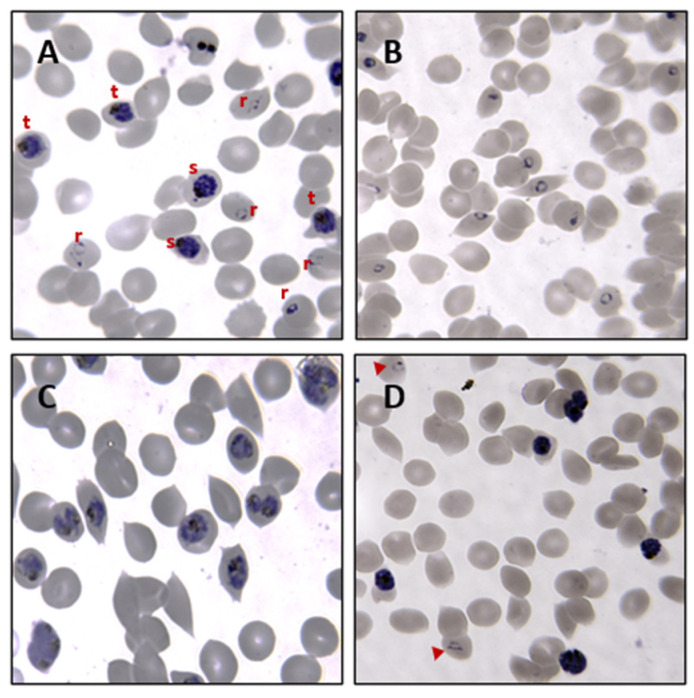
Giemsa-stained smears of *P. falciparum* iRBC cultures. (**A**) Asynchronous culture showing RBCs infected with ring forms (r) at different stages of development, trophozoites (t), and schizonts (s). The smear was made from a culture with 22% parasitemia. (**B**) Late rings at 20–22 h. (**C**) Late trophozoites at 36–38 h. (**D**) Mature schizonts at 44–46 h and two young rings at <6 h can be seen (red arrow heads). Images B, C, and D are of smears from the same culture with 15% parasitemia at the time of CCM harvest.

Before synchronization, *P. falciparum* cultures comprise iRBCs with ring, trophozoite, and schizont stages (Figure 6A). These are asynchronous cultures that will contain a mixed population of EVs from RBCs infected with all 3 asexual life stages of the parasite.

**Figure 7:**
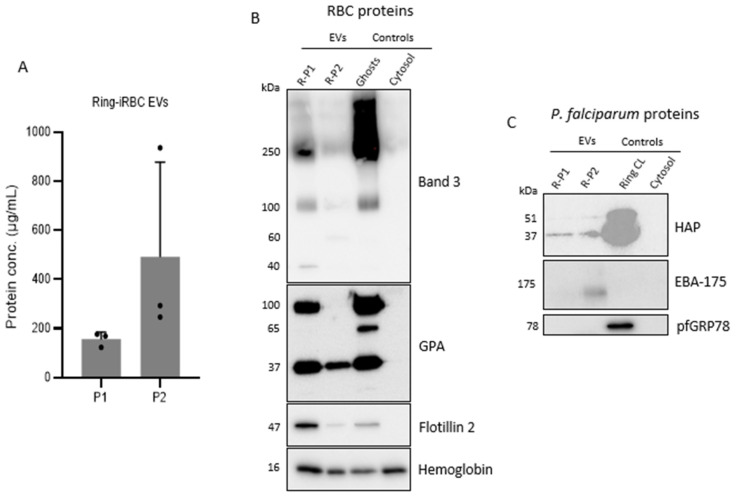
Two EV subtypes with differing protein concentration and composition are isolated using an optimized DC protocol. (**A**) The protein concentration of P2, as determined by a BCA protein assay, is significantly higher than that of P1; data show 3 biological replicates. (**B**,**C**) WBA of proteins in EVs. Here, 1 µg of protein for each EV and control was loaded onto pre-cast tris-glycine gels. SDS-PAGE was performed under reducing conditions except for EBA-175, which was performed under non-reducing conditions. Antibody dilutions for analyzed proteins were: band 3—1:5000; GPA, flotillin 2 and PfGRP78—1:1000; hemoglobin and HAP—1:2000; EBA-175—1:400. Molecular weights of identified proteins are shown to the left of the blots. Ghost membranes and cytosol of healthy RBCs, and cell lysates (CL) of rings obtained by saponin lysis of ring-iRBCs, are used as controls. (**B**) Band 3, GPA, and flotillin 2 are significantly enriched in P1 EVs. A 40-kDa band is detected in P1 while a 60-kDa band is detected slightly in P2. Two dimers of GPA are detected in P1, and a single low-molecular-weight dimer is detected in P2. The ~65 kDa GPA band in RBC ghosts is not detected in EVs. Flotillin 2 is strongly detected in P1 EVs relative to P2. Hemoglobin, which is a known component of RBC EVs, is detected in P1 and P2 iRBC-EVs. (**C**) The pro-enzyme (faint 51-kDa band) and mature form (37-kDa) of histoaspartic protease (HAP) is detected in malaria EVs. Erythrocyte binding antigen 175 (EBA-175) is detected faintly in P2 but not in P1 EVs, while glucose regulated protein 78 (PfGRP78) is not detected in either EV subtypes. R—Rings.

Following a combination of sorbitol and Percoll synchronization techniques, highly synchronous cultures of ring- and trophozoite-iRBCs at ≥95% synchrony and a narrow synchronization window of 4–6 h can be observed at the time of CCM harvest (Figure 6B,C). For the schizont stages, a synchrony of ≥90% is achievable; however, this is often lower at around 85%. This is because at the late schizont stage, a few very young rings (4–6 h post-invasion) can be observed (Figure 6D). As shown in Figure 7A, BCA protein assay of EV isolates from iRBC cultures typically reveals a higher protein concentration in P2 EVs (isolated at 100,000× *g* ultracentrifugation) than P1 EVs (isolated at 10,000× *g* centrifugation). Although determining the protein concentration of EV preparations gives a rough estimate of the amount of vesicles present in that preparation, it is often misleading as an overestimation due to the presence of co-isolating protein contaminants from the culture medium used to grow the EV-secreting cells [26,62]. Nevertheless, NTA shows that P2 contains significantly more particles than P1 (Appendix D).

Remarkably, P1 EVs are typically more enriched for the RBC EV markers band 3, GPA, and flotillin 2 compared to P2, and the WBA profile for specific proteins is generally different between P1 and P2 EVs across ring-, trophozoite-, and schizont iRBC-EVs [51]. The inverse relationship between total protein concentration (and particle concentration) and target protein enrichment in P1 and P2 may be attributed to higher amounts of co-isolating non-EV proteins in P2 than P1. Importantly, this inverse relationship suggests to us that P1 EVs, while fewer in particle number, load more cargo than P2 EVs and are, therefore, denser, pelleting at lower centrifugation speeds. Figure 7B shows representative data using EVs from ring-iRBCs (additional experimental replicates can be found in Appendix E). The band 3 antibody used in this protocol (see Section 2.1, Materials) recognizes the 100-kDa protein, a 250-kDa aggregate, as well as 60-kDa, 40-kDa, and 20-kDa peptides of the cytoplasmic domain of band 3. In our optimization studies (five biological replicates, two of which are shown in Appendix E), the 250-kDa, and 100-kDa bands are either faintly detected or not detected at all in the P2 EVs. In contrast, these are clearly detected in P1 EVs, suggesting that band 3 is a suitable marker for this EV subtype.

It is important to note that fragmentation of the 40-kDa peptide is the result of band 3 cleavage on the cytoplasmic surface of the RBCs while cleavage of the 60-kDa peptide occurs on the extracellular surface. The differential expression of these peptides in P1 and P2 malaria EVs (Figure 7B) may be an indication of EV subtypes with different biogenetic pathways in iRBCs that are successfully separated by this DC protocol. We, therefore, propose that this protocol is useful in isolating EVs for the investigation of their biogenesis in malaria infection. Our proposition is further substantiated by the differential expression of the 90–100 kDa GPA homodimer in P1 and P2 EVs (Figure 7B). This pattern is a consistent observation. Dimerization of GPA is a highly specific process [70] and may be implicated in EV biogenesis. The 37-kDa GPA dimer is strongly detected in both P1 and P2 EVs, suggesting that GPA is a valid general marker for malaria EVs. The ~65-kDa band in the RBC ghost membranes is presumably a GPA/GPB heterodimer [71] and is not detected in malaria EVs. In the data presented here, flotillin 2, which is a lipid raft-associated protein and bonafide EV marker [26], is enriched in ring-iRBC P1 but has a weak signal in the P2 EVs (Figure 7B). We have previously reported a stronger signal in this EV subtype [51].

Our observation from multiple biological replicates is that band 3 (Appendix E) and flotillin 2 are invariably more enriched in ring- and schizont-iRBC P1 EVs than the corresponding P2 EVs, but their expression in both trophozoite-iRBC P1 and P2 EVs is inconsistent. We recommend that for studies including EVs from trophozoite iRBCs, band 3 and/or flotillin 2 be used in conjunction with GPA and one or more other lipid raft-associated proteins for their characterization.

The host RBC membrane proteins analyzed in this study are known EV markers [26,72,73]. These are useful markers of malaria-derived EVs, as the parasite is intraerythrocytic, and malaria-derived EVs invariably bud off the RBC membrane [47]. However, establishing the specificity of *Plasmodium* proteins as EV markers is an ongoing process. We propose that analyzing non-secretory parasite proteins in EVs by WBA is helpful in validating the presence of parasite-derived EVs, as well as in studies of EV biogenesis and cargo sorting in *P. falciparum* [51]. This is because non-secretory parasite proteins are localized to the parasite [74]. A suitable candidate is HAP (histoaspartic protease, also known as plasmepsin III), which is a *P. falciparum* protein that is localized to the digestive vacuole and is predicted to not be secreted into the host RBC [74]. HAP is detected in malaria EVs as a proenzyme at 51 kDa and in its mature form at 37 kDa (Figure 7C and Appendix F). EBA-175 (erythrocyte binding antigen-175) is detected in ring-iRBC P2 EVs (Figure 7C) and, alongside its counterpart EBA-181, has been reported in WBA of EVs in another study [14]. Erythrocyte binding proteins are non-secretory [74] microneme proteins in the extracellular merozoite stage of *P. falciparum* and are mainly associated with the mature schizont intracellular asexual life stages [75,76]. Therefore, despite being a useful non-secretory parasite protein for WBA of malaria EVs, defining this protein as a potential parasite EV marker (particularly for schizont-iRBC EVs) requires further investigation and validation. The endoplasmic reticulum protein PfGRP78 (glucose regulated protein, also known as BiP) is not detected in malaria EVs (Figure 7C), and this is in line with published reports [14]. BiP may be analyzed to define a protein profile for a malaria EV population under investigation [26].

Although technically demanding and lengthy, the differential centrifugation EV isolation technique/protocol described here is not capital intensive, as the equipment required is often readily available to research labs. More importantly, EVs recovered using this protocol are a reliable source material for discovery malaria EV research, such as the study of malaria EV biology and biogenesis. This protocol, therefore, lays a good foundation for continued functional EV studies. The protocol is reproducible, yielding malaria EV subtypes with defined characteristics from the different life-stage infected RBCs. Such a protocol is a step forward towards standardizing malaria EV isolation and is important for intra- and inter-study comparisons, validating novel isolation techniques for malaria EVs, as well as validating findings of malaria EV studies.

## 5. Reagent Setup

- Incomplete medium

Use 500 mL RPMI 1640 (with L-glutamine, 25 mM HEPES, and sodium bicarbonate) and 200 µL (20 µg/ mL) gentamicin. Store at 4 °C for up to 4 weeks.

- Complete medium

Use 500 mL RPMI 1640 (with L-glutamine, 25 mM HEPES and sodium bicarbonate), 20 µg/ mL gentamicin, 100 µM hypoxanthine, and 5 mg/ mL AlbuMAX I. Store at 4 °C for up to 4 weeks.

- Thawing solution I: Sterile 12% NaCl (*w/v*)

Use 12 g NaCl and 100 mL distilled water. Pass through a 0.2 µm filter. Store at room temperature for 3–6 months.

- Thawing solution II: Sterile 1.67% NaCl (*w/v*)

Use 1.67 g NaCl, 100 mL distilled water. Pass through a 0.2 µm filter. Store at room temperature for 3–6 months.

- Thawing solution III: Sterile 0.9% NaCl + 0.2% D-glucose (*w/v*)

Use 0.9 g NaCl, 0.2 g D-glucose, and 100 mL distilled water. Pass through a 0.2 µm filter. Store at room temperature for 3–6 months.

- 10% Giemsa working solution (*v/v*)

Use 5 mL Giemsa modified stain solution and 45 mL distilled water. Prepare fresh.

- 5% sorbitol solution (*w/v*)

Use 5 g D-sorbitol and 100 mL distilled water. Pass through a 0.2 µm filter. Store at room temperature for 3–6 months.

- 90% Percoll solution (*v/v*)

Use 90 mL Percoll and 10 mL 10× PBS. Pass through a 0.2 µm filter. Store at 4 °C for up to a year.

- 65% Percoll solution (*v/v*)

Use 6.5 mL 90% Percoll and 2.5 mL incomplete medium. Pass through a 0.2 µm filter.

- 35% Percoll solution (*v/v*)

Use 3.5 mL 90% Percoll and 5.5 mL incomplete medium. Pass through a 0.2 µm filter.

- 10× tris-buffered saline (TBS)

Use 24 g Tris and 88 g NaCl. Adjust final volume of distilled water to 1 L

- 10× PBS

Use 80 g (1.37 M) NaCl, 2 g (27 mM) KCl, 17.8 g (100 mM) Na_2_HPO_4_, and 2.4 g (18 mM) KH_2_PO_4_. Adjust pH to 7.2. Adjust final volume of distilled water to 1 L. Pass through a 0.2 µm filter. Store at 4 °C for up to a year.

- 10% saponin solution (*w/v*)

Use 5 g saponin and 50 mL PBS. Dissolve at 37 °C. Pass through a 0.2 µm filter. Store at 4 °C for up to a year.

- 5 mM sodium phosphate buffer

Use 20.2 g (0.07541 M) Na_2_HPO_4_ and 3.4 g (0.02459 M) NaH_2_HPO_4_. Adjust pH to 8.0. Adjust final volume of distilled water to 1 L. Pass through a 0.2 µm filter. Store at 4 °C for up to a year.

- 6× Laemmli’s buffer

Use 1.2 g SDS, 6 mg bromophenol blue, 4.7 mL glycerol, 2.1 mL distilled water, and 1.2 mL of 0.5 M Tris. Dissolve all ingredients. Add 0.93 g DTT only for reducing conditions. Aliquot and store at −20 °C for up to 6 months. Do not re-freeze thawed aliquots.

- 10× SDS running buffer

Use 29 g Tris, 144 g glycine, and 10 g SDS. Adjust the final volume of distilled water to 1 L.

- 1× transfer buffer

Use 3.03 g Tris, 14.4 g glycine, 200 mL methanol, and 800 mL distilled water.

- 5% tris-buffered saline milk-tween 20 (5% TBSM-T; blocking buffer)

Use 200 mL 1× TBS, 200 µL Tween-20, and 10 g skim milk powder. Prepare fresh and store at 4 °C during use.

- 0.5% tris-buffered saline milk-tween 20 (0.5% TBSM-T; incubation buffer)

Use 200 mL 1× TBS, 200 µL Tween-20, and 1 g skim milk powder. Prepare fresh and store at 4 °C during use.

- 0.1% tris-buffered saline-tween 20 (0.1% TBS-T; wash buffer)

Use 200 mL 1× TBS and 200 µL Tween-20. Prepare fresh and store at 4 °C during use.

## Figures and Tables

**Figure A1:**
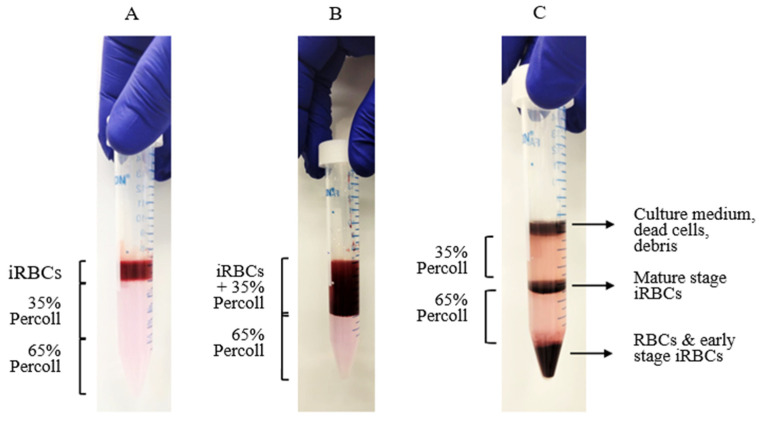
The use of 2 Percoll concentrations (65% and 35%) allows debris to be collected at the interface between the culture medium and 35% Percoll [67] (Figure 4). Ideally, the iRBC pellet should be layered on top of the 35% Percoll solution with a clear interface (**A**). We observed, however, that in some cases, layering the RBC pellet over 35% Percoll with a clear interface cannot be achieved, and the cells mix with the 35% Percoll (**B**). This may be due to the density of the donor blood cells. In such a situation, proceeding with centrifugation immediately still separates the mature-stage iRBCs from dead cells and debris (**C**), yielding synchronized parasites when cells are returned to culture. An alternative protocol, where cells are layered over a single Percoll concentration (60 to 70%), may also be used to achieve synchronization.

**Figure A2:**
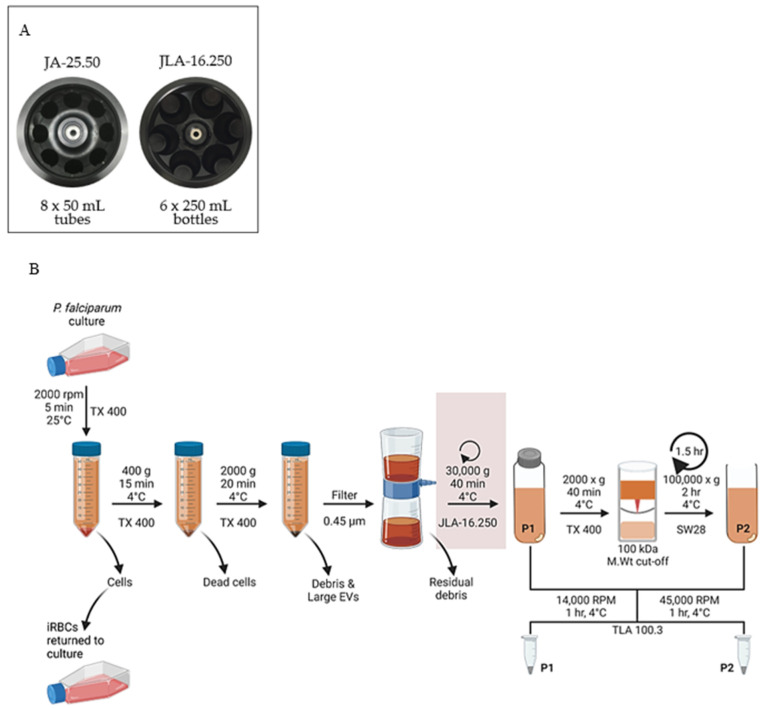
(related to Figure 5). To accommodate larger starting volumes of CCM and promote ease of handling to isolate the first malaria EV pellet, Intellifuge software was used to transfer the protocol for the JA-25.50 rotor, which has a nominal capacity of 400 mL (**A**, left), to the JLA-16.250 rotor with a nominal capacity of 1500 mL (**A**, right). EVs are isolated using the JLA-16.250 rotor by centrifugation at a higher speed of ~30,000× *g* and shorter run time of 40 min (**B**, shaded area) compared to centrifugation at 10,000× *g* for 1 h in a JA-25.50 rotor to obtain qualitatively and quantitatively comparable EV isolates.

**Figure A3:**
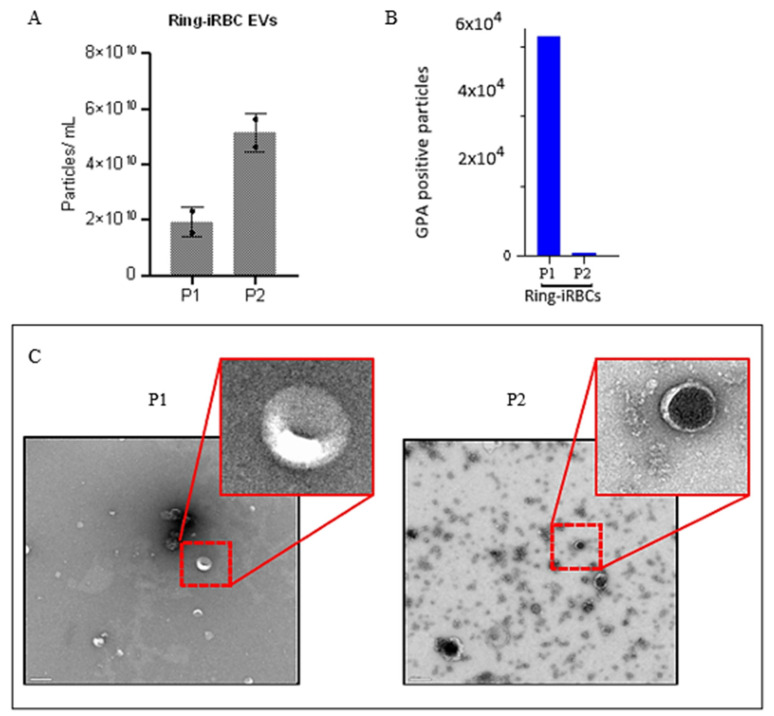
NTA (**A**), FC (**B**) and TEM (**C**). NTA of ring-iRBC-EVs shows that the particle concentration of P2 is significantly more than P1; data show 2 biological replicates (**A**). We have previously reported similar findings for trophozoite- and schizont-iRBC EVs [51]. Conversely, FC of ring-iRBC EVs with glycophorin A (GPA) showed that GPA positive particles were more abundant in P1 EVs than in P2 EVs; these data are from a single experimental run (**B**); however, the results mirror those of WBA, whereby several RBC membrane proteins that serve as EV markers are consistently detected in higher abundance in P1 compared to P2 EVs (see Figure 7B and Appendix E). The higher particle concentration of P2 as seen in (**A**) is likely due to higher amounts of co-isolating non-EV proteins in P2 than P1. This is evident in the TEM images, whereby background protein aggregates are observed in P2 EVs compared to P1; the large images are wide-field representations of at least 4 captures of each EV sample, while the insets show enlarged single EVs (small red boxes) (**C**). TEM reveals that malaria-derived EVs are generally small-sized at ≤200 nm in diameter. Scale bar: 200 nm at 23,000×.

**Figure A4:**
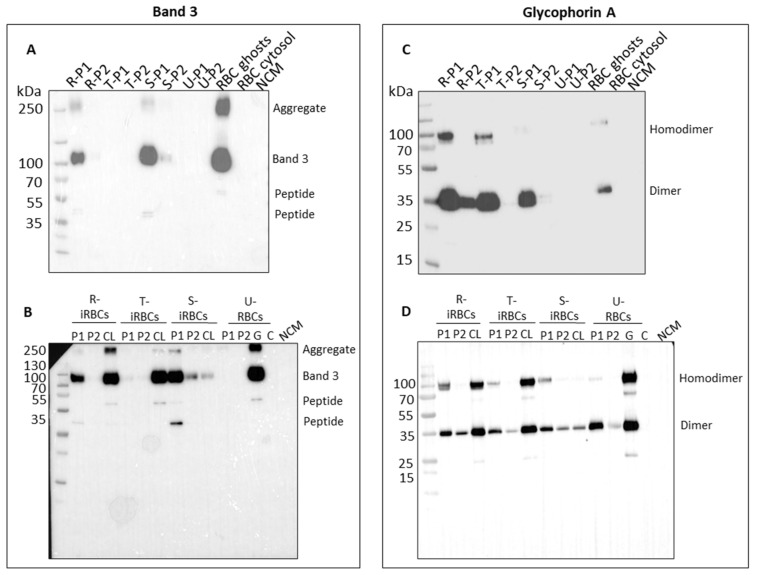
WBA of band 3 and glycophorin A (GPA). P1 and P2 EVs isolated from ring-, trophozoite-, and schizont-stage iRBCs show similar results from different biological replicates (data show 2 biological replicates), indicating reproducibility of the EV isolation protocol described; this is related to Figure 7B. Here, 1 µg of protein for each EV and control was loaded onto pre-cast tris-glycine gels. SDS-PAGE was performed under reducing conditions. Antibody dilutions for analyzed proteins were band 3—1:5000; GPA—1:1000. Molecular weights of identified proteins are shown to the left of the blots. As a control, EVs were isolated from parasite-free RBCs (U—uninfected RBCs) maintained under the same culture conditions as iRBCs and included in downstream analyses to determine baseline vesiculation in uninfected RBCs (uRBCs). Ghost membranes (G) and cytosol (C) of healthy RBCs are used as controls. In (**C**,**D**), cell lysates (CL) of parasites obtained by saponin lysis of iRBCs are used as additional controls. Band 3 (**A**,**B**) and GPA (**C**,**D**) are significantly enriched in P1 EVs. Notably, 2 dimers of GPA are detected in P1 EVs, and a single lower molecular weight dimer is detected in P2. R—Rings, T—trophozoites, S—schizonts.

**Figure A5:**
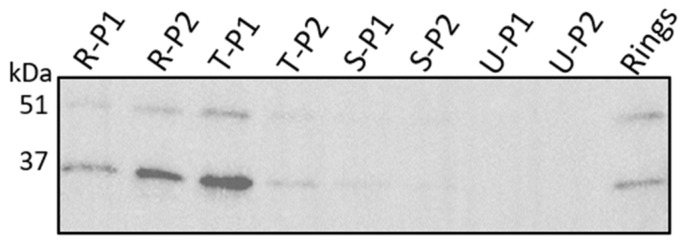
HAP is detected at 51 kDa and 37 kDa in EVs isolated from ring-, trophozoite-, and schizont-iRBCs. The observed bands in schizont P1 and P2 are faint compared to ring and trophozoite EVs (related to Figure 7C). The last column (rings) contains the lysate of *P. falciparum* rings from ring-infected RBCs and serves as a positive control for HAP. R—rings, T—trophozoites, S—schizonts, U—uninfected RBCs.

## Data Availability

Data contained within the article.

## Appendix A

**Table A1:** Modified *P. falciparum* culture conditions to increase EV yield.

P. falciparum Culture Parameter	Typical Condition	Modified Condition for EV Isolation
Parasitemia	5%	≥10%
Hematocrit	4%	≤2%
Age of RBCs	Up to 28 days after collection	Up to 14 days after collection
RBC preparation	Not usually stated	Leukoreduced

**Table A2:** Reportable parameters in malaria EV studies.

Parameter		Details
RBC for parasite culture	Blood group	A, B, AB, O
	Processing method	Leukoreduced or non-leukoreduced
	Storage duration from time of collection	1 to 14 days post collection
Culture conditions	Nutrient source	AlbuMAX I, AlbuMAX II, or serum
	Culture medium	Brand, supplements
	Positioning *	Stationary or shaking
	Hematocrit	≤2% or up to 4%
	Parasitemia	Low or high; less than or greater than 10%
CCM harvest	Intervals of harvest	e.g., 12 or 24 h post invasion
	Parasite life stage	Ring-, trophozoite-, or schizont CCM
	Volume	Exact volume stated
CCM storage	Temperature	4 °C, −80 °C
	Duration	Number of days, weeks, or months
EV Isolation	Concentration step	Vivacell units or other means used to concentrate CCM
	Rotor	Type of rotor and centrifuge
	Centrifugation speeds	RPM, RCF

* Culture flasks in this study were kept stationary.
